# Robust estimation of sulcal morphology

**DOI:** 10.1186/s40708-019-0098-1

**Published:** 2019-06-11

**Authors:** Christopher R. Madan

**Affiliations:** School of Psychology, University of Nottingham, Nottingham, NG7 2RD UK

**Keywords:** Sulcal width, Sulcal depth, Age, Cortical structure, Atrophy, Gyrification, Cerebral sulci

## Abstract

While it is well established that cortical morphology differs in relation to a variety of inter-individual factors, it is often characterized using estimates of volume, thickness, surface area, or gyrification. Here we developed a computational approach for estimating sulcal width and depth that relies on cortical surface reconstructions output by FreeSurfer. While other approaches for estimating sulcal morphology exist, studies often require the use of multiple brain morphology programs that have been shown to differ in their approaches to localize sulcal landmarks, yielding morphological estimates based on inconsistent boundaries. To demonstrate the approach, sulcal morphology was estimated in three large sample of adults across the lifespan, in relation to aging. A fourth sample is additionally used to estimate test–retest reliability of the approach. This toolbox is now made freely available as supplemental to this paper: https://cmadan.github.io/calcSulc/.

## Introduction

**Fig. 1:**
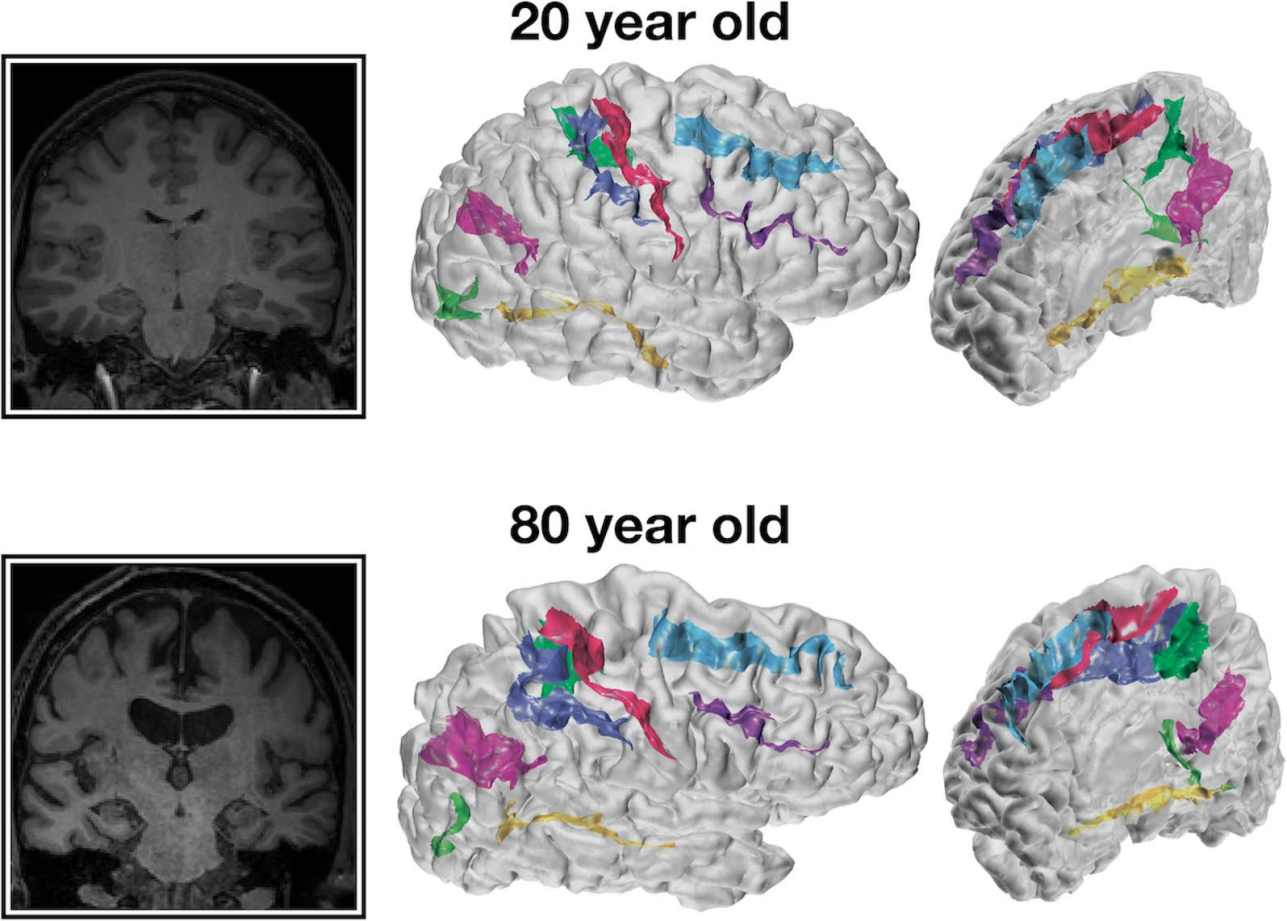
Representative coronal slices and cortical surfaces with sulcal identification for 20- and 80-year-old individuals

Cortical structure differs between individuals. It is well known that cortical thickness generally decreases with age [1–11]; however, a more visually prominent difference is the widening of sulci, sometimes described as ‘sulcal prominence’ [12–17]. In the literature, this measure has been referred to using a variety of names, including sulcal width, span, dilation, and enlargement, as well as fold opening. With respect to aging and brain morphology, sulcal width has been assessed qualitatively by clinicians as an index of cortical atrophy [12, 13, 15, 16, 18, 19]. An illustration of age-related differences in sulcal morphology is shown in Fig. 1.

Using quantitative approaches, sulcal width has been shown to increase with age [20–23] likely relating to subsequent findings of age-related decreases in cortical gyrification [2, 5–7, 24]. Sulcal widening has also been shown to be associated with decreases in cognitive abilities [25] and physical activity [26]. With respect to clinical conditions, increased sulcal width has been found in dementia patients relative to healthy controls [27–33], as well as with schizophrenia patients [34–36] and mood disorders [37].

One of the most common programs for conducting cortical surface analyses is FreeSurfer [38]. Unfortunately, though FreeSurfer reconstructs cortical surfaces, it does not estimate sulcal width or depth, leading researchers to use FreeSurfer along with another surface analysis program, BrainVISA [39–42], to characterize cortical thickness along with sulcal morphology (e.g, [22, 25, 26, 43, 44]). While this combination allows for the estimation of sulcal morphology in addition to standard measures such as cortical thickness, FreeSurfer and BrainVISA rely on different anatomical landmarks [45] which can yield differences in their resulting cortical surface reconstructions [46]. Admittedly, determining the boundaries for an individual sulcus and incorporating individual cortical variability is difficult [45, 47–52]. While an enumerate amount of other methods have already been proposed to identify and characterize sulcal morphology (e.g., [53–74]), ultimately these all are again using different landmarks than FreeSurfer uses for cortical parcellations (i.e., volume, thickness, surface area, gyrification). Note that, though FreeSurfer itself does compute sulcal maps, these are computed as normalized depths, not in real-world units (e.g., [75]); furthermore, these are also independent of sulcal width information.

Here we describe a procedure for estimating sulcal morphology and report age-related differences in sulcal width and depth using three large samples of adults across the lifespan: two of these datasets are from Western samples, Dallas Lifespan Brain Study (DLBS) and Open Access Series of Imaging Studies (OASIS), as well as one East Asian sample, Southwest University Adult Lifespan (SALD), as potential differences between populations have been relatively understudied [76, 77]. To further validate the method, test–retest reliability was also assessed using a sample of young adults who were scanned ten times within the span of a month [78, 79]. All four of these datasets are open-access and have sufficient sample sizes to be suitable for brain morphology research [77]. This procedure has been implemented as a MATLAB toolbox, calcSulc, that calculates sulcal morphology—both width and depth—using files generated as part of the standard FreeSurfer cortical reconstruction and parcellation pipeline. This toolbox is now made freely available as supplemental to this paper: https://cmadan.github.io/calcSulc/.

## Materials and methods

**Fig. 2:**
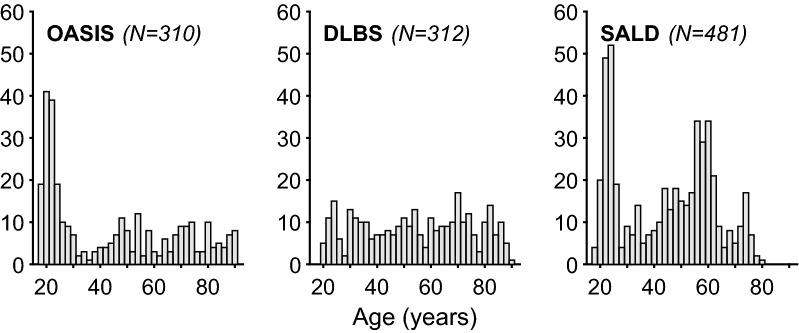
Histogram of age distribution for the three aging datasets: OASIS, DLBS, and SALD, only for participants included in the sulcal morphology analyses. Each bar corresponds to a 2-year age-range bin

### Datasets

#### OASIS

This dataset consisted of 314 healthy adults (196 females), aged 18–94 (see Fig. 2), from the Open Access Series of Imaging Studies (OASIS) cross-sectional dataset (http://www.oasis-brains.org) [80]. Participants were recruited from a database of individuals who had (a) previously participated in MRI studies at Washington University, (b) were part of the Washington University Community, or (c) were from the longitudinal pool of the Washington University Alzheimer Disease Research Center. Participants were screened for neurological and psychiatric issues; the Mini-Mental State Examination (MMSE) and Clinical Dementia Rating (CDR) were administered to participants aged 60 and older. To only include healthy adults, participants with a CDR above zero were excluded; all remaining participants scored 25 or above on the MMSE. Multiple T1 volumes were acquired using a Siemens Vision 1.5 T with a MPRAGE sequence; only the first volume was used here. Scan parameters were: TR $=$ 9.7 ms; TE $=$ 4.0 ms; $\hbox {flip angle} = 10^{\circ }$; $\hbox {voxel size}=1.25 \times 1 \times 1 \, \hbox {mm}$. Age-related comparisons for volumetric and fractal dimensionality measures from the OASIS dataset were previously reported [7, 81, 82].^1^

#### DLBS

This dataset consisted of 315 healthy adults (198 females), aged 20–89 (see Fig. 2), from wave 1 of the Dallas Lifespan Brain Study (DLBS), made available through the International Neuroimaging Data-sharing Initiative (INDI) [83] and hosted on the Neuroimaging Informatics Tools and Resources Clearinghouse (NITRC) [84] (http://fcon_1000.projects.nitrc.org/indi/retro/dlbs.html). Participants were screened for neurological and psychiatric issues. No participants in this dataset were excluded *a priori*. All participants scored 26 or above on the MMSE. T1 volumes were acquired using a Philips Achieva 3 T with a MPRAGE sequence. Scan parameters were: TR $=$ 8.1 ms; TE $=$ 3.7 ms; $\hbox {flip angle} = 12 ^{\circ }$; $\hbox {voxel size} = 1 \times 1 \times 1 \, \hbox {mm}$. See Kennedy et al. [85] and Chan et al. [86] for further details about the dataset. Age-related comparisons for volumetric and fractal dimensionality measures from the DLBS dataset were previously reported [7, 81, 82].$^{1}$

#### SALD

This dataset consisted of 483 healthy adults (303 females), aged 19–80 (see Fig. 2), from the Southwest University Adult Lifespan Dataset (SALD) [87], also made available through INDI and hosted on NITRC (http://fcon_1000.projects.nitrc.org/indi/retro/sald.html). No participants in this dataset were excluded *a priori*. T1 volumes were acquired using a Siemens Trio 3 T with a MPRAGE sequence. Scan parameters were: TR $=$ 1.9 s; TE $=$ 2.52 ms; $\hbox {flip angle} = 9 ^{\circ }$; $\hbox {voxel size} = 1 \times 1 \times 1 \, \hbox {mm}$.

#### CCBD

This dataset consisted of 30 healthy adults (15 females), aged 20–30, from the Center for Cognition and Brain Disorders (CCBD) at Hangzhou Normal University [78]. Each participant was scanned for 10 sessions, occurring 2–3 days apart over a 1-month period. No participants in this dataset were excluded *a priori*. T1 volumes were acquired using a SCANNER with a FSPGR sequence . Scan parameters were: TR $=$ 8.06 ms; TE $=$ 3.1 ms; $\hbox {flip angle} = 8 ^{\circ }$; $\hbox {voxel size}: 1 \times 1 \times 1 \, \hbox {mm}$. This dataset is included as part of the Consortium for Reliability and Reproducibility (CoRR) [88] as HNU1. Test–retest comparisons for volumetric and fractal dimensionality measures from the CCBD dataset were previously reported [79].$^{1}$

### Procedure

Data were analyzed using FreeSurfer v6.0 (https://surfer.nmr.mgh.harvard.edu) on a machine running Red Hat Enterprise Linux (RHEL) v7.4. FreeSurfer was used to automatically volumetrically segment and parcellate cortical and subcortical structures from the T1-weighted images [38, 89] FreeSurfer’s standard pipeline was used (i.e., recon-all). No manual edits were made to the surface meshes, but surfaces were visually inspected. Cortical thickness is calculated as the distance between the white matter surface (white–gray interface) and pial surface (gray-CSF interface). Gyrification was also calculated using FreeSurfer, as described in Schaer et al. [90]. Cortical regions were parcellated based on the Destrieux et al. [91] atlas, also part of the standard FreeSurfer analysis pipeline.

## Calculation

Here we outline a novel, simple yet robust, automated approach for estimating sulcal width and depth, based on intermediate files generated as part of the standard FreeSurfer analysis pipeline. This procedure and functionality has been implemented in an accompanying MATLAB toolbox, calcSulc. The toolbox is supplemental material to this paper and is made freely available: https://cmadan.github.io/calcSulc/.

**Fig. 3:**
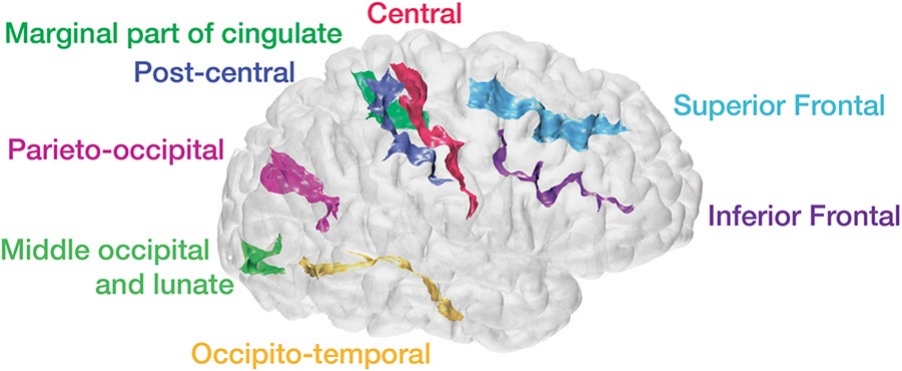
Example cortical surface with estimated sulci identified and labeled

**Fig. 4:**
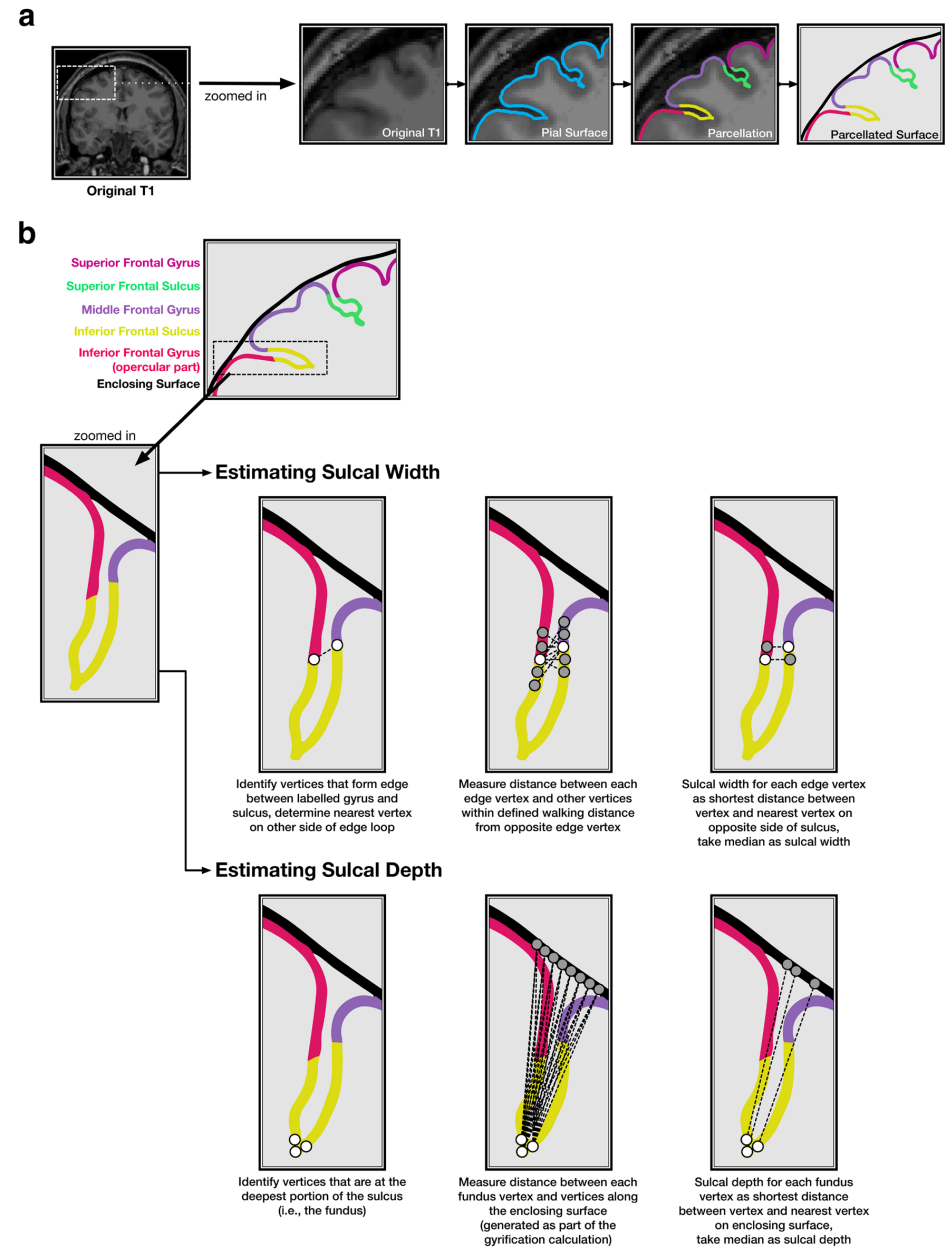
Illustration of the sulcal morphology method. **a** Cortical surface estimation and sulcal identification, as output from FreeSurfer. **b** Sulcal width and depth estimation procedure. Note that the surface mesh and estimation algorithm use many more vertices than shown here

For each individual sulcus (for each hemisphere and participant), the following approach was used to characterize the sulcal morphology. The procedure has been validated and is supported for the following sulci: central, post-central, superior frontal, inferior frontal, parieto-occipital, occipito-temporal, middle occipital and lunate, and marginal part of the cingulate (S_central, S_postcentral, S_front_sup, S_front_inf, S_parieto_occipital, S_oc-temp_med&Lingual, S_oc_middle&Lunatus, S_cingul-Marginalis). All of the sulci are labeled in Fig. 3. An overview of the approach is illustrated in Fig. 4.

First the pial surface and Destrieux et al. [91] parcellation labels were read into MATLAB by using the FreeSurfer-MATLAB toolbox provided alongside FreeSurfer (calcSulc_load), this consists of the ?h.pial (FreeSurfer cortical surface mesh) ?h.aparc.a2009s.annot (FreeSurfer parcellation annotation) files. Using this, the faces associated with the individual sulcus were isolated as a 3D mesh (calcSulc_isolate).

The width of each sulcus (calcSulc_width) was calculated by determining which vertices lay on the boundary of the sulcus and the adjacent gyrus. An iterative procedure was then used to determine the ‘chain’ of edges that would form a contiguous edge loop that encircle the sulcal region (calcSulc_getEdgeLoop). This provided an exhaustive list of all vertices that were mid-way between the peak of the respective adjacent gyri and depth of the sulcus itself. For each vertex in this edge-loop, the nearest point in 3D space that was *not* neighboring in the loop was determined, with the goal of finding the nearest vertex in the edge that was on the opposite side of the sulcus—i.e., a line between these two vertices would ‘bridge’ across the sulcus. Since these nearest vertices in the edge loop are not necessarily the nearest vertex along the opposite sulcus wall, an exhaustive search (walk) was performed, moving up to a 4 edges from the initially determined nearest vertex (configurable as options.setWidthWalk). The sulcal width was then taken as the median of these distances that bridged across the sulcus (see Fig. 4).

The depth of each sulcus (calcSulc_depth) additionally used FreeSurfer’s sulcal maps (?h.sulc) to determine the relative inflections in the surface mesh, which would be in alignment with the gyral crown. The deepest points of the sulcus, i.e., the sulcal fundus, were taken as the 100 vertices within the sulcus with the lowest values in the sulcal map. For these 100 vertices, the shortest (i.e., Euclidean) distance to the smoothed enclosing surface was calculated (generated by FreeSurfer’s built-in gyrification analysis [?h.pial-outer-smoothed], [90]), and the median of these was then taken as the sulcal depth. While the use of a Euclidean distance here underestimates the true sulcal depth, it is nonetheless robust (as demonstrated in the present work) and does not markedly differ from other algorithmic approaches for estimating sulcal depth for much of the cortex (see [74] for a comparison).

Sulcal morphology, width and depth, was estimated for eight major sulci in each hemisphere: central, post-central, superior frontal, inferior frontal, parieto-occipital, occipito-temporal, middle occipital and lunate, and marginal part of the cingulate. Preliminary analyses additionally included superior and inferior temporal sulci and intraparietal sulcus, but these were removed from further analysis when the sulci width estimation was found to fail to determine a closed boundary edge-loop at an unacceptable rate ($>10\%$) for at least one hemisphere. This edge boundary determination failed when parcellated regions were labeled by FreeSurfer to comprise at least two discontinuous regions, such that they could not be identified using a single edge loop. Nonetheless, sulcal measures failed to be estimated for some participants, resulting in final samples of 310 adults from the OASIS dataset, 312 adults from the DLBS dataset, 481 adults from the SALD dataset, and 30 adults from the CCBD dataset (see Fig. 2).

### Test–retest reliability

Test–retest reliability was assessed as intraclass correlation coefficient (*ICC*), which can be used to quantify the relationship between multiple measurements [79, 92–98]. McGraw and Wong [99] provide a comprehensive review of the various *ICC* formulas and their applicability to different research questions. *ICC* was calculated as the one-way random effects model for the consistency of single measurements, i.e., *ICC*(1, 1). As a general guideline, *ICC* values between .75 and 1.00 are considered ‘excellent,’ .60–.74 is ‘good,’ .40–.59 is ‘fair,’ and below .40 is ‘poor’ [100].

## Results and discussion

### Age-related differences in sulcal morphology

**Fig. 5:**
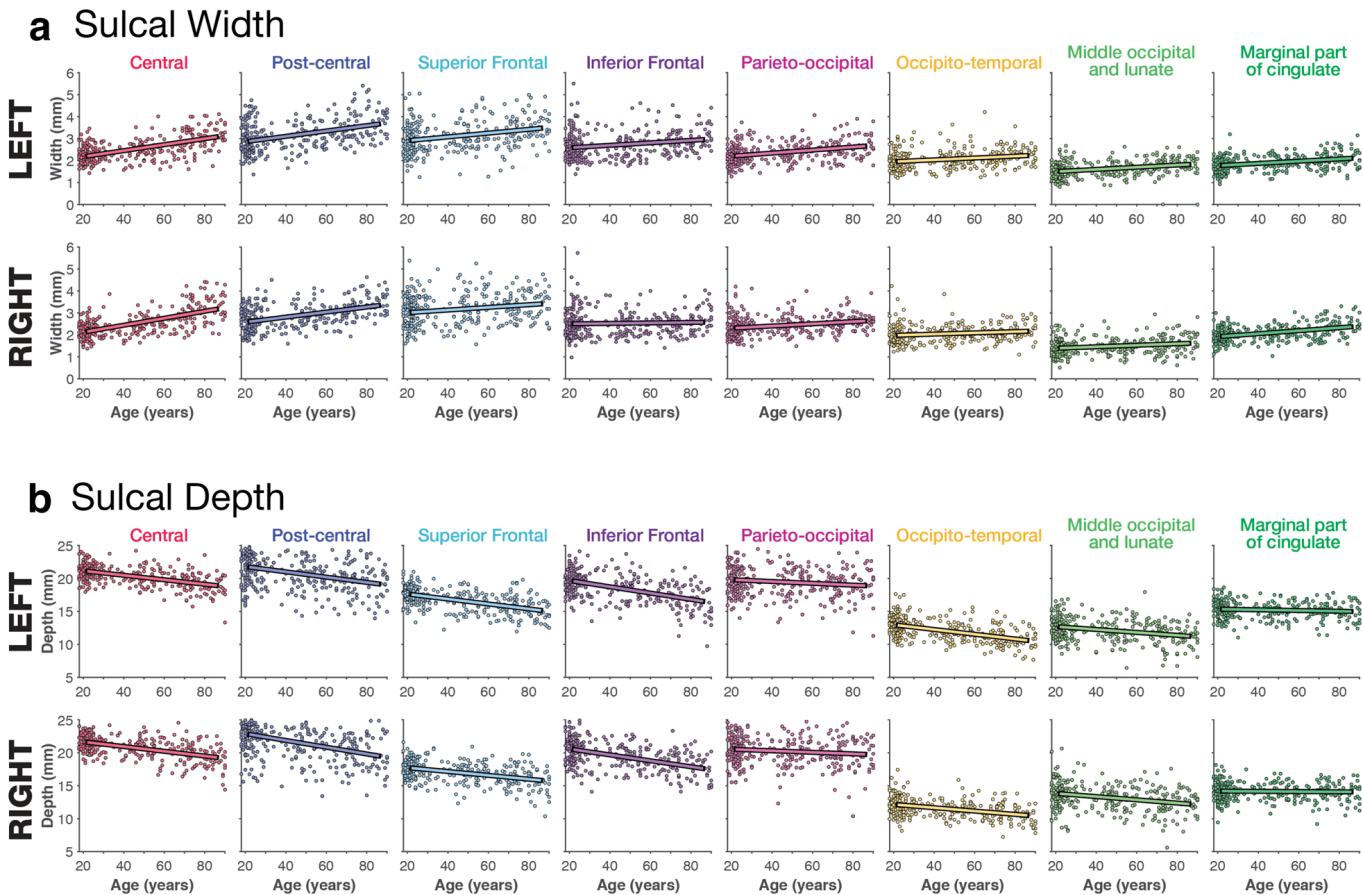
Relationship between **a** sulcal depth and **b** width for each of the sulci examined, based on the OASIS dataset

Scatter plots showing the relationships between each individual sulcal width and depth and age, for the OASIS dataset, are shown in Fig. 5; the corresponding correlations for all datasets are shown in Tables 1 and 2. The width and depth of the central and post-central sulci appear to be particularly correlated with age, with wider and shallower sulci in older adults. Age-related differences in sulcal width and depth and generally present in other sulci as well, but are generally weaker.

Age-related relationships for each sulcus were relatively consistent between the two Western lifespan datasets (OASIS and DLBS), but age-related differences in sulcal width (but not depth) were markedly weaker in the East Asian lifespan dataset (SALD). This finding will need to be studied further, but may be related to gross differences in anatomical structure [101–103]—and motivates the need to aging in samples that vary in ethnicity/race and are otherwise not of a so-called WEIRD (Western, Educated, Industrialized, Rich, and Democratic) demographic [77]. Additionally, there did not appear to be a significant influence of field strength (i.e., 1.5 T for the OASIS dataset vs. 3 T for the DLBS dataset) on estimates of sulcal morphology. Importantly, test–retest reliability, *ICC*(1, 1), was particularly good for the sulcal depth across individual sulci.

**Table 1:** Correlations between sulcal width and age for each sulci and hemisphere, for each of the three lifespan datasets examined

Sulci name	FreeSurfer label^a^	Hemi.	OASIS	DLBS	SALD	CCBD	
			*r* (age)	*r* (age)	*r* (age)	ICC(1,1)	95% CI of ICC
Central	S_central	L	.586	.486	.322	.858	[ 0.785, 0.918]
		R	.632	.523	.294	.842	[ 0.764, 0.908]
Post-central	S_postcentral	L	.413	.391	.198	.764	[ 0.660, 0.858]
		R	.460	.436	.213	.864	[ 0.794, 0.922]
Superior Frontal	S_front_sup	L	.281	.421	.055	.797	[ 0.703, 0.880]
		R	.205	.291	.035	.843	[ 0.764, 0.909]
Inferior frontal	S_front_inf	L	.217	.323	− .037	.775	[ 0.675, 0.865]
		R	.043	.222	− .036	.831	[ 0.748, 0.901]
Parieto-occipital	S_parieto_occipital	L	.348	.279	.145	.616	[ 0.486, 0.753]
		R	.257	.357	.213	.682	[ 0.561, 0.802]
Occipito-temporal	S_oc-temp_med&Lingual	L	.227	.270	− .055	.660	[ 0.535, 0.786]
		R	.168	.189	.017	.692	[ 0.572, 0.808]
Middle occipital and lunate	S_oc_middle&Lunatus	L	.306	.271	.145	.605	[ 0.474, 0.744]
		R	.212	.177	.023	.625	[ 0.496, 0.760]
Marginal part of cingulate	S_cingul-Marginalis	L	.340	.275	.075	.783	[ 0.685, 0.871]
		R	.430	.382	.161	.757	[ 0.651, 0.853]
Mean			.636	.592	.227	.907	[ 0.856, 0.947]

Test–retest reliability, *ICC*(1, 1), is also included from the CCBD dataset

^a^FreeSurfer labels in version 6.0; labels are named slightly different in version 5.3. *ICC* values between .75 and 1.00 are considered ‘excellent,’ .60–.74 is ‘good,’ .40–.59 is ‘fair,’ and below .40 is ‘poor’ [100]

**Table 2:** Correlations between sulcal depth and age for each sulci and hemisphere, for each of the three lifespan datasets examined

Sulci name	FreeSurfer label^a^	Hemi.	OASIS	DLBS	SALD	CCBD	
			*r* (age)	*r* (age)	*r* (age)	ICC (1,1)	95% CI of ICC
Central	S_central	L	− .517	− .205	− .346	.848	[ 0.772, 0.912]
		R	− .505	− .256	− .348	.860	[ 0.789, 0.919]
Post-central	S_postcentral	L	− .371	− .264	− .268	.965	[ 0.944, 0.981]
		R	− .436	− .246	− .330	.890	[ 0.831, 0.937]
Superior frontal	S_front_sup	L	− .523	− .454	− .397	.899	[ 0.844, 0.943]
		R	− .413	− .465	− .444	.886	[ 0.825, 0.935]
Inferior frontal	S_front_inf	L	− .517	− .490	− .491	.932	[ 0.893, 0.962]
		R	− .496	− .480	− .490	.915	[ 0.868, 0.952]
Parieto-occipital	S_parieto_occipital	L	− .145	− .093	− .241	.979	[ 0.966, 0.989]
		R	− .124	.059	− .229	.970	[ 0.952, 0.984]
Occipito-temporal	S_oc-temp_med&Lingual	L	− .509	− .323	− .263	.953	[ 0.926, 0.974]
		R	− .404	− .316	− .281	.913	[ 0.864, 0.951]
Middle occipital and lunate	S_oc_middle&Lunatus	L	− .290	− .167	− .150	.949	[ 0.919, 0.972]
		R	− .288	− .120	− .132	.922	[ 0.879, 0.956]
Marginal part of cingulate	S_cingul-Marginalis	L	− .092	− .035	− .268	.952	[ 0.925, 0.974]
		R	− .032	− .017	− .156	.918	[ 0.872, 0.954]
Mean			− .465	− .645	− .600	.972	[ 0.955, 0.985]

Test–retest reliability, *ICC*(1, 1), is also included from the CCBD dataset

^a^FreeSurfer labels in version 6.0; labels are named slightly different in version 5.3. *ICC* values between .75 and 1.00 are considered ‘excellent,’ .60–.74 is ‘good,’ .40–.59 is ‘fair,’ and below .40 is ‘poor’ [100]

To obtain a coarse summary measure across sulci, we averaged the sulcal width across the 16 individual sulci for each individual, and with each dataset, and examined the relationship between mean sulcal width with age. These correlations, shown in Table 1, indicate that the mean sulcal width was generally a better indicator of age-related differences in sulcal morphology than individual sulci, and had increased test–retest reliability. Mean sulcal depth was similarly more sensitive to age-related differences than for an individual sulcus (e.g., it is unclear why the relationship between age and width of the central sulcus differed between samples) and the magnitude of this relationship was more consistent across datasets. Reliability was even higher for mean sulcal depth than mean sulcal width.

### Comparison with other age-related structural differences

Within each dataset, mean sulcal depth and width correlated with age, as shown in Tables 1 and 2. Of course, other measures of brain morphology also differ with age, such as mean (global) cortical thickness [OASIS: $r(308)=-.793$, $p<.001$; DLBS: $r(310)=-.759$, $p<.001$; SALD: $r(479)=-.642$, $p<.001$]. and volume of the third ventricle (ICV-corrected) [OASIS: $r(308)=.665$, $p<.001$; DLBS: $r(310)=.677$, $p<.001$; SALD: $r(479)=.328$, $p<.001$]. Previous studies have demonstrated that both of these measures are robust estimates of age-related differences in brain structure [1–6, 8–11, 81, 104].

To test if these mean sulcal measures served as distinct measures of age-related differences in brain morphology, beyond those provided by other measures, such as mean cortical thickness and volume of the third ventricle, we conducted partial correlations that controlled for these two other measures of age-related atrophy. Mean sulcal width [OASIS: $r_p(306)=.188$, $p<.001$; DLBS: $r_p(308)=.177$, $p=.002$; SALD: $r(477)=.003$, $p=.96$] and depth [OASIS: $r_p(306)=-.443$, $p<.001$; DLBS: $r_p(308)=-.397$, $p<.001$; SALD: $r_p(477)=-.534$, $p<.001$] both explained unique variance in relation to age. Thus, even though more established measures of age-related differences in brain morphology were replicated here, the additional sulcal measures captured aspects of aging that are not accounted for by these extant measures, indicating that these sulcal measures are worth pursuing further and are not redundant with other measures of brain structure. Providing additional support for this, mean sulcal width and depth were only weakly related to each other [OASIS: $r(308)=-.192$, $p<.001$; DLBS: $r(310)=.092$, $p=.104$; SALD: $r(479)=.119$, $p=.009$].

As with the individual sulci measures, we did observe a difference between samples where some age-related measures were less sensitive in the East Asian lifespan sample (SALD), here in the ventricle volume correlation and the unsurprisingly weaker age relationship in the partial correlation using sulcal width. These sample differences are puzzling, though there is a general correspondence between the two Western samples. Given that much of the literature is also based on Western samples, we think further research with East Asian samples, and particularly comparing samples with the same analysis pipeline, is necessary to shed further light on this initial finding.

## Conclusion

Differences in sulcal width and depth are quite visually prominent, but are not often quantified when examining individual differences in cortical structure. Here we examined age-related differences in both sulcal measures as a proof-of-principle to demonstrate the utility of the calcSulc toolbox that accompanies this paper and is designed to closely compliments the standard FreeSurfer pipeline. This allows for the additional measurement of sulcal morphology, to add to the extant measures of brain morphology such as cortical thickness, area, and gyrification. Critically, this approach uses the same landmarks and boundaries as in the Destrieux et al. [91] parcellation atlas, in contrast to all previous approaches to characterize sulcal features. This toolbox is now made freely available as supplemental to this paper: https://cmadan.github.io/calcSulc/.

Using this approach, here we demonstrate age-related differences in sulcal width and depth, as well as high test–retest reliability. Since individual differences in sulcal morphology are sufficiently distinct from those characterized by other brain morphology measures, this approach should complement extant work of investigating factors that influence brain morphology, e.g., see Fig. 3 of Madan and Kensinger [7]. Given the flexibility in the methodological approach, these measures can be readily applied to other samples after being initially processed with FreeSurfer .

## Footnotes

1 Note that analyses reported in these previous papers were based on preprocessing in FreeSurfer v5.3.0, rather than FreeSurfer v6.0.
